# Uncovering the Prokaryotic Diversity of Hypersaline Soils of Odiel Saltmarshes Natural Area Through Metagenome-Assembled Genomes

**DOI:** 10.3390/microorganisms14020489

**Published:** 2026-02-18

**Authors:** Cristina Galisteo, Fernando Puente-Sánchez, Rafael R. de la Haba, Stefan Bertilsson, Antonio Ventosa, Cristina Sánchez-Porro

**Affiliations:** 1 Department of Microbiology and Parasitology, Faculty of Pharmacy, University of Sevilla, 41002 Sevilla, Spain; crigalgomez@us.es (C.G.); rrh@us.es (R.R.d.l.H.); ventosa@us.es (A.V.); 2 Department of Aquatic Sciences and Assessment, Swedish University of Agricultural Sciences, 75651 Uppsala, Sweden; fernando.puente.sanchez@slu.se (F.P.-S.); stefan.bertilsson@slu.se (S.B.)

**Keywords:** hypersaline soils, prokaryotic diversity, *Candidatus*, heavy metal tolerance, osmoregulation

## Abstract

The hypersaline soils of the Odiel Saltmarshes Natural Area in Southwest Spain harbor highly diverse microbial communities adapted to extreme conditions. However, their genomic diversity remains largely unexplored. In addition to high salinity, these soils are contaminated with heavy metals, creating a hostile environment of great interest for studying extremophilic microorganisms and their metabolic adaptations. This study aims to characterize the uncovered prokaryotic taxa as *Candidatus* species inhabiting the hypersaline soils of the Odiel Saltmarshes, based on their metagenomic assembled genomic sequences. The reconstructed genomes were assessed for quality based on completeness and contamination thresholds and subsequently taxonomically classified. Comparative genomic analysis of six high-quality MAGs revealed key metabolic traits related to survival under extreme salinity and heavy metal conditions. The findings provide new insights about microbial diversity of hypersaline environments and expand the catalog of known prokaryotic genomes. Detailed characterization of six novel *Candidatus* taxa highlights the unique adaptations of these microorganisms, enhancing our understanding of life in extreme habitats.

## 1. Introduction

In recent years, hypersaline soils have been a focal point in prokaryotic biodiversity studies due to the alarming increase in salinity levels in agricultural lands [1,2,3]. Most of those studies have relied on culture-independent techniques such as shotgun metagenomics or, for the most part, targeted amplicon sequencing. These approaches have revealed that the dominant prokaryotic taxa in these environments include members of the archaeal phylum *Methanobacteriota* and bacterial phyla *Pseudomonadota*, *Balneolota*, *Bacteroidota*, and *Rhodothermota*. Culture-dependent approaches have also been informative in retrieving and characterizing both abundant and less prevalent novel species, including *Chromohalobacter sarecensis* [4], *Echinicola soli* [5], and multiple species within the haloarchaeal genus *Haloarcula* [6,7,8,9], among others [10,11,12,13,14,15]. However, a substantial proportion of the prokaryotic diversity in hypersaline terrestrial environments remains uncharacterized [16,17,18,19] as most viable cells are subject to the Great Plate Counting Anomaly. Currently, the development of sequencing methodologies and bioinformatic tools grant a new approach to biodiversity research and discovery of microbial species. To date, approximately 40% of GTDB (Genome Taxonomy Database) species are constituted exclusively by Metagenomic Assembled Genomes (MAGs) [20]. It allows us the possibility to study the phylogenomic relationship, metabolism, and ecological impact of species that are not successfully grown under laboratory conditions.

The microbiome of hypersaline soils of the Odiel Saltmarshes Natural Area, settled between the Odiel and Tinto rivers in Southwest Spain, has been a recent focus of interest by both culture-dependent and independent techniques [12,13,14,,21,22,23,24,25]. These studies include the description of uncovered archaeal and bacterial species such as *Aquibacillus salsiterrae* [12], *Haloarcula saliterrae* and *H. onubensis* [7,9], *Fodinibius salsisoli* [14], *Pseudidiomarina terrestris* [13], or the rare genus *Terrihalobacillus* [12]. The Odiel Saltmarshes Natural Area has suffered from nonregulated industry and mining in the past, responsible for the high concentration of salt and heavy metal [21,26,27,28,29,30]. Consequently, it endures electroconductivity values above the 15 mS/cm threshold (World Reference Base, WRB) and high concentrations of arsenic, cadmium, copper, lead and zinc [21,29,30].

In these habitats, extreme salinity and heavy metal concentrations act as powerful selective filters, driving evolutionary and adaptive processes that are reflected in the specialized functional traits encoded within the genomes of the resident microbial communities [16,21].

Due to our interest in the complexity of the prokaryotic life in this environment, we carried out a study of its diversity based on shogun sequencing of 18 samples from three different areas (named as Area 1, 2 and 3) at two consecutive years (2020 and 2021) [21]. Our discoveries revealed *Metanobacteriota* as the archaeal phylum in mayor representation, and *Pseudomonadota*, *Bacteroidota*, *Gemmatimonadota*, and *Balneolota* as the main phyla within the domain *Bacteria*, with approximately 10% of the bacterial reads unable to be attributed to any known phylum. Most sequences could not be identified to lower taxonomic ranks, but the archaeal families *Halorubraceae*, *Haloferacaceae*, *Haloarculaceae* and *Halobactericeae*, and the bacterial family *Balneolaceae* were the most representative groups at that taxonomic level [21]. Furthermore, some of the most abundant phyla in this particular environment, such as *Nitrospinota*, *Gemmatimonadota* or *Balneolota* are groups with few representative isolates. Concerning the strategies for extreme conditions tolerance, we detected genomic sequences associated with arsenic mobilization and metalloid efflux systems. The sequences related to the domain *Archaea* exhibited a low isoelectric point associated with “salt-in” osmoregulation strategy as well as genes related to de novo biosynthesis of osmolytes. Moreover, “salt-in” and “salt-out” mechanisms could be identified in bacterial proteomes. On the other hand, no association could be established between the prokaryotic community and fluctuations of the physicochemical parameters within samples [21]. While the general taxonomic and functional profiles of these metagenomic datasets were previously reported [21], the present study applies a new bioinformatic approach focused on high-resolution genome binning to reconstruct and characterize high-quality Metagenome-Assembled Genomes (MAGs) of the most relevant uncultured taxa.

Given the rich and yet uncovered microbial diversity of extreme environments, particularly, the hypersaline soils of the Odiel Saltmarshes Natural Area, we aim to explore the unknown taxa-based MAGs of high quality. Beyond taxonomic inventories, the reconstructed population genomes would shed light on the metabolism and environmental adaptations of uncultured abundant species, including their tolerance strategies to extreme concentrations of salt and heavy metals.

## 2. Materials and Methods

### 2.1. Metagenomic Data

The present research constitutes an extension of a previous study about the microbial community inhabiting the hypersaline soils of the Odiel Saltmarshes Natural Area, which exhibited a great proportion of unknown taxa [21] of which we aim to shed on some light. For it, we studied 18 soils samples collected in three sampling sites (1, 2 and 3), with three replicates for each sample (A, B and C), during the years 2020 and 2021 (Table 1). DNA extraction, quality control, library preparation and short-read sequencing is detailed in Galisteo et al. [21], along with raw reads preprocessing and assembly. Shortly, raw sequences were filtered with ‘PRINSEQ’ v.0.20.3 [31], for which ‘N’ strings at the terminal positions were trimmed, reads with entropy <70 and length <60 bp were removed and singletons were discarded. Assembly was performed within the ‘SqueezeMeta’ v.1.6.0 [32].

### 2.2. MAGs Reconstruction and Classification

MAGs were reconstructed independently for the 18 metagenomic databases using ‘MetaBAT’ v. 2.12.1 [33] and ‘MaxBin’ v. 2.2.6 [34], and integrated with ‘DAS tool’ v. 1.1.1 [35]. The completeness and contamination of the final MAGs were calculated with ‘CheckM’ v. 1.0.11 [36]. The relative abundance for the total sequencing effort was normalized using FPM (Features Per Million), allowing reliable comparisons within and between samples, as well as between genes of different sizes. Coding sequences were predicted by ‘Prodigal’ v. 2.6.3 [37] and their translated sequences were annotated against the KEGG database [38] using ‘DIAMOND’ v. 2.0.14.152 [39]. All previously mentioned tools were implemented into the automated pipeline ‘SqueezeMeta’ v.1.6.0 [32]. The taxonomy of the MAGs was assessed using the ‘GTDB-Tk’ v. 2.0.0 tool [40] against GTDB database v. 207.0 [20].

MAGs were classified following the Minimum Information about a Metagenome-Assembled Genome (MIMAG) criteria [41], based on the percentage of completeness and contamination in: (i) high quality, HQ (>90% completeness, <5% contamination); (ii) medium quality, MQ (≥50% completeness, <10% contamination); and (iii) low quality, LQ (<50% completeness, <10% contamination). For the best represented bacterial family among reconstructed sequences, HQ and MQ MAGs were clustered into 95% ANI metagenomic Operative Taxonomic Units (mOTUs) considering their Nucleotide Identity and quality parameters using ‘mOTUlizer’ v.0.3.2 [42].

### 2.3. Phylotaxonomic Analysis

The status of each MAG within their respective taxonomic group was established using Overall Genome Relatedness Indexes (OGRIs), following the proposed minimal standards for prokaryotic taxonomy [43]. Average Amino acid Identity (AAI) for genus delineation was computed using the ‘Enveomic’ toolbox [44]. ‘OAU’ software v. 1.2 [45] calculated Average Nucleotide Identity for orthologous sequences (orthoANI) for species differentiation. To infer the placement of the MAG to their closely related species, phylogenomic trees based on the core proteome were built. First, orthologous amino acid sequences were identified by ‘BLASTp’ v. 2.2.28+ and extracted by Markov Cluster Algorithm implemented in the ‘Enveomics’ toolbox [44]. The alignment was performed with ‘Muscle’ v. 3.8.31 [46]. Approximately maximum-likelihood algorithm implemented in ‘FastTreeMP’ v. 2.1.8 [47] calculated the phylogeny of the concatenated orthologous proteins considering the Jones-Taylor-Thornton model of amino acid evolution [48]. The reliability of each node was established with the Shimodaira-Hasegawa test [49]. The final imaging of the trees was plotted with ’gitana’ script [50] (https://github.com/cristinagalisteo/gitana, accessed on 1 November 2025).

## 3. Results

Results related to the taxonomic and functional profiles at contigs level of the 18 metagenomic dataset were previously reported and discussed by Galisteo et al. [21]. Information about the sequencing features is detailed in Table S1. In this study, we focused on the characterization of several new *Candidatus* species based on their reconstructed genomic sequences from the metagenomes.

### 3.1. Reconstruction of Genomic Sequences

A total of 4718 MAGs were reconstructed. For each of the 18 metagenomic datasets, the number of MAGs varied between 353 and 205. Following the MIMAG criteria, we obtained 11 HQ MAGs, 273 MQ MAGs and 2919 LQ MAGs (Table 2). The remainder of the MAGs fell outside these categories.

### 3.2. Taxonomic Assignment

**Figure 1:**
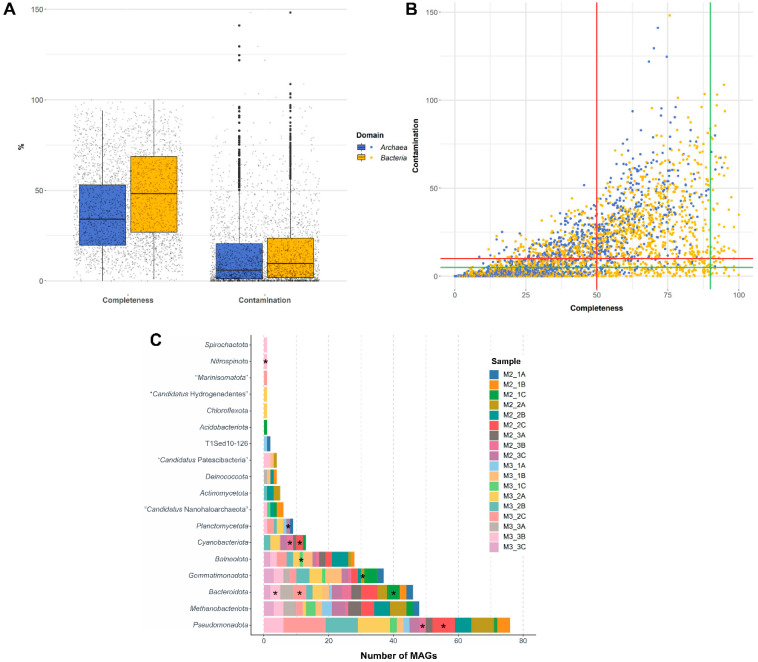
(**A**) Boxplot and (**B**) distribution of completeness and contamination percentages for the 3164 MAGs identified to domain level. The green lines define the values considered to correspond to high-quality bins, and the red lines define the medium-quality bins. (**C**) Number of high- and medium-quality MAG for the 18 shotgun metagenomes according to their assigned phylum. Asterisk (*) indicates high-quality MAGs.

The taxonomic annotation of the MAGs was performed against the GTDB database [20]. Out of the 4718 MAGs, 3164 could be classified to at least domain rank using ‘GTDB-Tk’ (option ‘classify’). The relatively balanced distribution between domains *Archaea* and *Bacteria* previously observed in the contig sequences of the 18 metagenomes [21] was also maintained among MAGs. In total, 1388 (29.4%) MAGs were identified as *Archaea* and 1776 (37.6%) MAGs as *Bacteria*. The archaeal MAGs were less complete, but also slightly less contaminated (Figure 1A). All 11 HQ MAGs, as well as most of the MQ MAGs, were affiliated with the domain *Bacteria* (Figure 1B). The low representation of archaeal taxa within the best quality MAGs could be explained by the prevailing population structure within this domain, where most of the archaeal populations in the environment under study fall within class *Halobacteriales* and genera with a high number of described species, such as *Halorubrum* (>50 species) or *Haloarcula* (>30 species) [21]. Thus, the similar G+C content and oligonucleotide frequencies, parameters used by the binning programs, might obscure the feature-based assembly [51].

#### 3.2.1. Minor Phyla Represented Among the High and Medium Quality MAGs

Our previous study identified more than 100 phyla using assembled sequences (contigs) of each metagenome [21]. Here, the HQ and MQ MAGs were affiliated with 18 of those phyla. Among them, *Pseudomonadota* was the phylum with the highest number of identified HQ and MQ MAGs (76 MAGs), followed by *Methanobacteriota* (48 MAGs), *Bacteroidota* (46 MAGs), *Gemmatimonadota* (37 MAGs), and *Balneolota* (28 MAGs) (Figure 1C). These results are consistent with the previous studies on the predominant phyla inhabiting the hypersaline soils of the Odiel Saltmarshes Natural Area [21,22] and other geographically distant hypersaline terrestrial environments such as the Tibetan plateau and the Atacama Desert [1,16,17,18,21,52,53]. One or more HQ MAGs were reconstructed for each of these taxa, with the exception of archaeal phylum *Methanobacteriota* (Figure 1C). The top-quality MAG within this *Methanobacteriota* group belonged to the haloarchaeal genus *Halorubrum* (91.62% completeness; 5.69% contamination) followed by members of the genera *Halobaculum* and *Halomarina* (83.49–83.48% completeness; 0.26–8.12% contamination), within the order *Halobacteriales*. Notably, six MAGs were affiliated with the minor archaeal phylum “*Ca*. Nanohaloarchaeota”, surpassing the number of reconstructed sequences of other predominant prokaryotic phyla in the hypersaline soils of the Odiel Saltmarshes Natural Area [21], i.e., *Actinomycetota* (5 MAGs), *Deinococcota* (4 MAGs), and *Chloroflexota* (1 MAG) (Figure 1C). It is worth noting that some MAGs were related to phyla that lack cultured representatives, i.e., “*Ca*. Patescibacteria” (4 MAGs), T1Sed10-126 (2 MAGs), “*Ca*. Hydrogenedentota” (1 MAG) and “*Ca*. Marinisomatota” (1 MAG), or with very few cultured representatives, i.e., *Nitrospinota* (1 HQ MAG) [36,45]. Other MQ MAGs were reconstructed for the phyla *Spirochaeotota* (1 MAG) and *Acidobacteriota* (1 MAG). None of the MAGs identified as members of the domain *Archaea* met the adopted high-quality standards (Figure 1B; Table S2).

#### 3.2.2. The *Balneolaceae* Family as the Best Represented Among the MAGs

Of the 284 HQ and MQ MAGs, only 47 could not be identified at the family level (41 belonging to the domain *Bacteria* and 6 to the *Archaea*). Among those, the family most frequently assigned was *Balneolaceae*, followed by the haloarchaeal families *Haloarculaceae* and *Haloferacaceae*, as well as the bacterial families *Cyclobacteriaceae* and *Wenzhouxiangellaceae*. On the other hand, a large proportion of families were represented by a single MAG, mostly families with invalidly published names according to the International Code of Nomenclature of Prokaryotes (Figure S1).

At the genus level, 73 MAGs were affiliated with 30 different bacterial genera: *Halalkalibaculum* (14 MAGs); *Fodinibius* (6 MAGs); *Erythrobacter* (5 MAGs); *Coleofasciculus* and *Halofilum* (4 MAGs each); *Alterinioella*, *Halomicronema*, *Owenweeksia*, *Salinibacter* and *Wenzhouxiangella* (3 MAGs each); *Gracilimonas*, *Nafulsella*, *Rivularia*, *Salinimicrobium* and *Silicimonas* (2 MAGs each); and *Alteriqipengyuania*, *Congregibacter*, *Cryomorpha*, *Fulvivirga*, *Gillisia*, *Halomonas*, *Marinobacter*, *Parvularcula*, *Pseudidiomarina*, *Pseudomonas*, *Rhodovibrio*, *Rubrimonas*, *Thiohalobacter*, *Thiohalophilus* and *Thiohalorhabdus* (1 MAG each). The two most represented genera among MAGs, *Halalkalibaculum* and *Fodinibius*, along with the genus *Gracilimonas*, are members of the family *Balneolaceae*, within the phylum *Balneolota*. This phylum has been previously identified as one of the main taxa in the soils under study [14,21,22], as well as in other hypersaline terrestrial environments [17,54]. These three genera (*Fodinibius*, *Gracilimonas* and *Halalkalibaculum*) represented 22 out of the 28 MAGs assigned to the family *Balneolaceae* (Figure S1), implying that there is diversity that has not been captured with cultivation-based methods in this family. The genus *Halalkalibaculum* has recently been described and, to date, it harbors a single species, *H. roseum* [55]. Thus, the large number of MAGs associated with this genus suggests that additional not-yet-described species are inhabiting the environment under study.

#### 3.2.3. The Best Quality MAGs Constitute Uncultured Species

As previously indicated, none of the MAGs identified as members of the domain *Archaea* met the adopted high-quality standards (Figure 1B; Table S2). The top-quality MAG within this group belonged to the genus *Halorubrum*, with 91.62% completeness and 5.69% contamination. Other good MAGs were 83.49–83.48% complete with 7.26–8.12% contamination and were taxonomically identified as members of the genera *Halobaculum* and *Halomarina*, within the order *Halobacteriales*.

Of the 11 HQ MAGs assembled during this study, it was possible to identify the top-quality MAG (99.95% completeness; 0.82% contamination) to species level, i.e., *Pseudomonas taetrolens*, within the phylum *Pseudomonadota* (Table S2). Its taxonomic affiliation was confirmed by orthoANI values above 97.4% for genomic sequences from *P. teatrolens* that are publicly available (GCA_001042915.1, GCA_900104825.1; GCA_900475285.1; GCA_900637735.1; GCA_044540965.1; GCA_963970545.1). This species is known for its production of lactobionic acid [56,57,58], a molecule of interest in the chemical and pharmaceutical industries [59].

To genus level, two HQ MAGs were affiliated with *Halomicronema* (family *Phormidesmiaceae*, phylum *Cyanobacteriota*) and *Halalkalibaculum* (designed as “g__YR4” in GTDB, family *Balneolaceae*, phylum *Balneolota*). Two HQ MAGs were identified to family rank as *Cyclobacteriaceae* (phylum *Bacteroidota*), and three additional HQ MAGs to families *Saprospiraceae* (phylum *Bacteroidota*), *Spirulaceae* (phylum *Cyanobacteriota*), and *Wenzhouxiangellaceae* (phylum *Pseudomonadota*), respectively. At higher taxonomic levels, a HQ MAG was assigned to the order *Longimicrobiales* (phylum *Gemmatimonadota*), which is currently represented by a single species, *Longimicrobium* terrae [15]. Lastly, two HQ MAGs could only be identified at the phylum level, particularly, phyla *Nitrospinota* and *Planctomycetota*, respectively (Table S2).

These results suggest that most of the HQ MAGs reconstructed in this study are constituting new uncharacterized taxa that have not yet been isolated. For instance, the phylum *Nitrospinota* comprises 218 representatives grouped into 44 genera according to GTDB [20] (last consulted 16 January 2025), where almost all of them are yet to be cultured. Previous studies have been successful in overcoming the challenge of cultivating representative strains of those not-yet-described halophilic bacterial taxa by the analysis of their metabolisms from their metagenomic reconstructed genome [60]. Thus, the high-quality features of the unknown MAGs would allow for further exploration of the biology and genome-encoded traits of these undescribed species.

Summarizing, the binning analysis succeeded in the reconstruction of ≥4700 MAGs, of which 284 showed high (11 MAGs) or acceptable (273 MAGs) quality in terms of completeness and contamination. Although the proportion of archaea and bacteria was similar, the MAGs assigned as *Bacteria* showed higher completeness, but also higher contamination than those assigned *Archaea*. The 284 MAGs that were further analyzed were distributed across 18 phyla, although most of them were comprised within phyla *Pseudomonadota*, *Methanobacteriota*, *Bacteroidota*, *Gemmatimonadota*, and *Balneolota*. Furthermore, *Balneolaceae* was the family with the largest number of identified MAGs (28). The lack of identification to low taxonomic level of 10 of 11 HQ MAGs suggests that they are representing novel taxa. Therefore, the Odiel Saltmarshes Natural Area maintain hitherto unknown microbial biodiversity.

In the following sections, the taxonomic position of selected HQ MAGs will be studied along with their putative metabolic activity encoded in the reconstructed genome sequences.

### 3.3. Phylogenomic Description of Novel Candidatus Taxa

We performed the phylotaxonomic study of the six HQ MAGs, specifically M2_3B_020, M2_2A_002, M3_3B_026, M2_1C_046, M3_2C_046 and M3_1C_030, that will be described as *Candidatus* species and/or genera.

MAGs M2_2C_043, M2_3B_044, M2_3C_069, M3_3B_085 and M2_2C_007 were excluded from this analysis. In the case of M2_2C_043, this MAG is related to the genus *Halomicronema*, but the lack of genomic data of the currently described species within this genus motivated its exclusion. MAG M2_3B_044 is related to the family *Spirulinaceae*. This family belongs to the phylum *Cyanobacteriota*, whose names are validly published under the International Code of Nomenclature for algae, fungi and plants (Botanical Code). Therefore, the current classification of this putative species is beyond the scope of this study and the field of knowledge of the authors. Similarly, we could not find enough evidence to establish the correct taxonomic position of M2_3C_069 and M3_3B_085 (phylum *Planctomycetota* and family *Saprospiraceae*, respectively), considering the available information of their closely related isolated species. Last, M2_2C_007, identified as *Pseudomonas teatrolens*, was already classified to species level and its taxonomic placement was verified by orthoANI percentages (previous section) so there is no need for a further exhaustive taxonomic study.

#### 3.3.1. Uncovering a New Species Within the Genus *Wenzhouxiangella*

**Figure 2:**
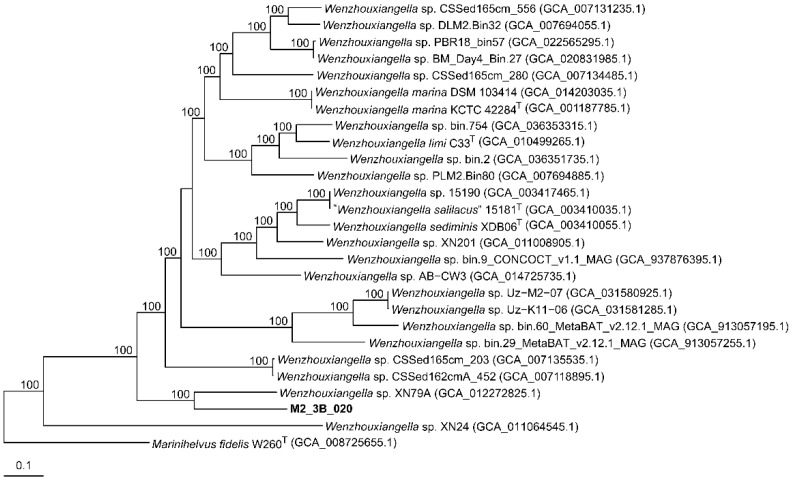
Approximately maximum-likelihood phylogenomic tree based on 586 concatenated orthologous translated proteins of M2_3B_020 and other representative sequences from the family *Wenzhouxiangellaceae*. Bootstrap values above 70% are shown above the branches. Bar, 0.1 substitutions per nucleotide position.

The HQ MAG M2_3B_020 (3,596,349 bp; 66.5 mol% G+C content) was identified as a member of the family *Wenzhouxiangellaceae*, within the phylum *Pseudomonadota* (Table S2). However, it could not be placed in any of the two currently described genera of this family, i.e., *Wenzhouxiangella* and *Marinihelvus* [61]. Given that only four isolate-derived genomes were available in public databases [62], we also included in our comparative genomic analysis MAG sequences identified as members of *Wenzhouxiangellaceae* with >90% completeness and <5% contamination. The orthoANI percentages establish that M2_3B_020 clusters with previously sequenced genomes and MAGs from the family *Wenzhouxiangellaceae*. The higher orthoANI result (75% against isolate *Wenzhouxiangella* sp. XN79A) was below the 95% cutoff for species delineation [63,64,65] (Figure S2), indicating that the genomic sequence M2_3B_020 does not resemble any of the currently known sequences. Additionally, AAI values ranged between 66.9% (*Wenzhouxiangella* sp. XN79A) and 57.0% (*Wenzhouxiangella* sp. bin.29_MetaBAT_v2.12.1_MAG) for sequences related to the genus *Wenzhouxiangella* (Figure S2). The only exception is against *Wenzhouxiangella* sp. XN24, but this genome showed values lower than those of the other species of *Wenzhouxiangella* against themselves (Figure S2), so they are not considered reliable enough. On the other hand, M2_3B_020 showed only 51.2% AAI similarity to *Marinohelvus* fidelis W260T, the sole representative of the genus *Marinohelvus*. Considering the genus delineation threshold of 62–72% AAI [66,67], M2_3B_020 appears to fall within the genus *Wenzhouxiangella*. Moreover, phylogenomic analysis based on 586 core genome translated proteins (Figure 2) reveals that M2_3B_020 is harbored within the *Wenzhouxiangella* cluster and shares a node (100% bootstrap) with an unknown species constituted by the isolate *Wenzhouxiangella* sp. XN79A. The large length of their branches (Figure 2), as well as their orthoANI percentage (Figure S2), indicate that M2_3B_020 represents a novel, uncultured species within *Wenzhouxiangella*. We propose the name “*Candidatus* Wenzhouxiangella saliterrae” sp. nov.

#### 3.3.2. Uncultured Novel Genus Within the Abundant Phylum *Gemmatimonadota*

The HQ MAG M2_2A_002 (3,347,599 bp; 70.4 mol% G+C) was identified as unknown taxon within the order *Longimicrobiales* (Table S2). This order is exclusively constituting the class *Longimicrobiia*, which, along with *Gemmatimonadales*, belong to the phylum *Gemmatimonadota*. In total, the phylum *Gemmatimonadota* harbors four genera and six species, one of them with nomenclature not validated according to the International Code of Nomenclature of Prokaryotes (ICNP). The only known species of the order *Longimicrobiales* is *Longimicrobium terrae* [15]. At present, it is an extremely unknown phylum with very few isolates. However, there are almost 4000 genomic sequences available at NCBI Genome database reconstructed from metagenomic analyses (last, 17 January 2025).

**Figure 3:**
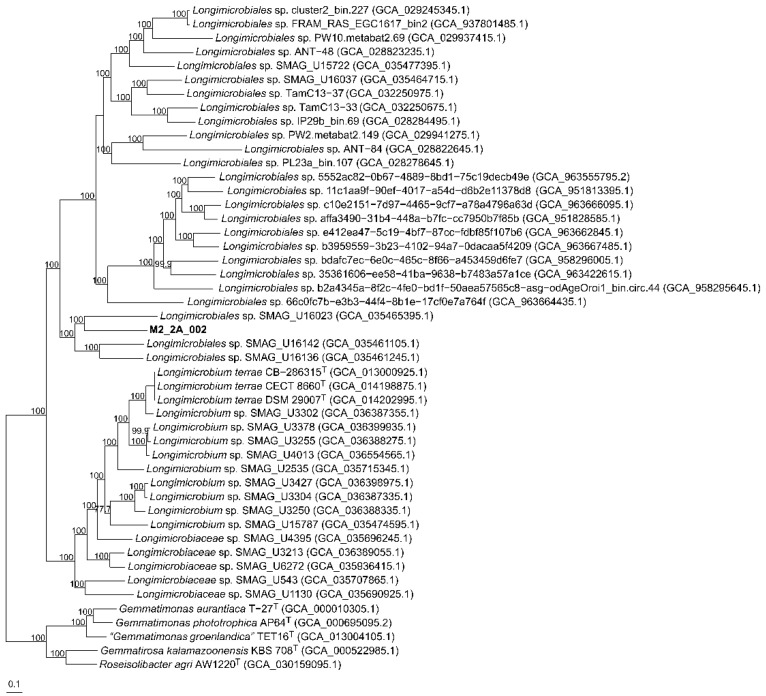
Approximately maximum-likelihood phylogenomic tree based on 223 concatenated orthologous translated proteins of M2_2A_002 and other representative sequences of the order *Longimicrobiales.* Bootstrap values above 70% are shown above the branches. Bar, 0.1 substitutions per nucleotide position.

The highest orthoANI percentage values (69%) were shown by M2_2A_002 with *Longimicrobiaceae* sp. SMAG_U3213, *Longimicrobiaceae* sp. SMAG_4395, *Longimicrobiaceae* sp. SMAG_U6272, *Longimicrobiaceae* sp. SMAG_U16023, *Longimicrobiaceae* sp. SMAG_U16136, *Longimicrobiaceae* sp. SMAG_U16142 and *Longimicrobium* sp. SMAG_U253 while lower values were observed for the rest of the genomes (Figure S3), indicating that the reconstructed genome M2_2A_002 does not correlate with any previously known sequences from species of the order *Longimicrobiales*. Furthermore, values are below the 74.0% ANI cutoff for genus demarcation proposed by Barco et al. [68]. In the case of AAI, the highest identity percentage for M2_2A_002 was 55.0% (*Longimicrobiales* sp. SMAG_U16142) (Figure S3), below the 65−72% for genus delineation [66,67]. The core genome based on 223 orthologous translated sequences of the 48 isolates and high-quality MAG sequences shows that M2_2A_002 clearly constitute a separate branch within the order *Longimicrobiales* (Figure 3). All parameters show that M2_2A_002 harbors the closest relationship with the members of the order *Longimicrobiales*, but a different species and genus than *Longimicrobium terrae*. Thus, we conclude that it constitutes a novel genus and species for which we propose the name “*Candidatus* Terrihalomicrobium hispanicum” gen. nov., sp. nov.

#### 3.3.3. Novel Genus in Uncultured Lineage of *Nitrospinota*

In the case of the HQ MAG M3_3B_026 (2,848,768 bp; 61.6 mol% G+C), it was identified to phylum level as part of *Nitrospinota* (Table S2). This taxonomic group is constituted by a single isolated genus, *Nitrospina*, and only two isolates, *Nitrospina gracilis* [69] and “*Nitrospina watsonii*” [70]. In addition, three more *Candidatus* genera with few described species have been described [61]. However, multiple genomic sequences have been reconstructed from metagenomic datasets, mostly from marine habitats [71].

**Figure 4:**
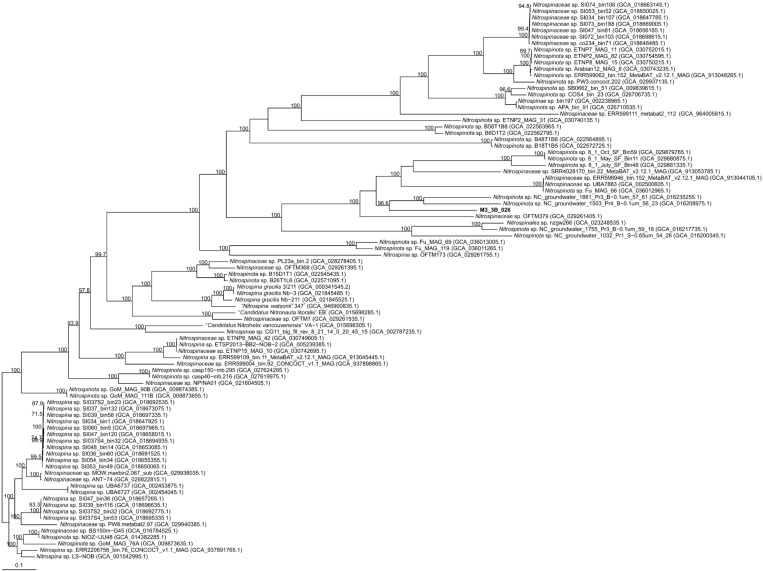
Approximately maximum-likelihood phylogenomic tree based on 98 concatenated orthologous translated proteins of M3_3B_026 and other representative sequences of the phylum *Nitrospinota*. Bootstrap values above 70% are shown above the branches. Bar, 0.1 substitutions per nucleotide position.

Considering the poor representation of the phylum *Nitrospinota*, MAGs with quality equal or above >90% completeness and <5% contamination identified as members of this group and available in the public databases were included in this analysis. Highest orthoANI percentage for M3_3B_026 (69%, *Nitrospinota* sp. NC_groundwater_1881_Pr3_B−0.1um_57_61) was clearly under the 95% and 74% for species and genera delineation [68], respectively (Figure S4). Similarly, the AAI values were equal or lower than 58.3% (the highest values were observed against *Nitrospinaceae* sp. OFTM379, *Nitrospinota* sp. NC_groundwater_1881_Pr3_B−0.1um_57_61 and *Nitrospinota* sp. NC_groundwater_1503_Pr4_B−0.1um_56_23) against all the sequences under study (Figure S4), clearly below the 65–72% threshold for genus delineation [66,67]. These results indicate that M3_3B_026 does not belong to any currently known species nor genera. Furthermore, this affirmation is supported by the core genome tree based on 98 orthologous translated proteins, where M3_3B_026 constitutes a single branch supported with a reliability of 96.6% (Figure 4). Hence, we suggest M3_3B_026 as a new genus and species within the phylum *Nitrospinota*, for which we propose the name “*Candidatus* Nitrohalomicrobium salipaludis” gen. nov., sp. nov.

#### 3.3.4. Identification of Two New Genera Within the Family *Cyclobacteriaceae*

**Figure 5:**
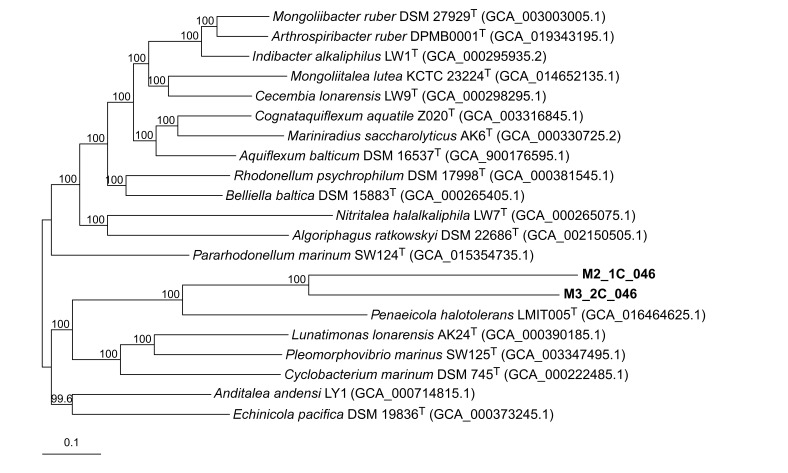
Approximately maximum-likelihood phylogenomic tree based on 906 concatenated orthologous translated proteins of M2_1C_046 and M3_2C_046 and other representative sequences of the family *Cyclobacteriaceae*. Bootstrap values above 70% are shown above the branches. Bar, 0.1 substitutions per nucleotide position.

The HQ MAGs M2_1C_046 (4,752,021 bp; 37.9 mol% G+C) and M3_2C_046 (6,246,477 bp; 40.1 mol% G+C) from this study were related to the family *Cyclobacteriaceae*, placed on the order *Cytophagales*, class *Cytophagia* and phylum *Bacteroidota* (Table S2). This family is constituted by 24 genera, of which 19 of them had the genome of a representative species of the genus available at public databases. Due to the good representation of the genome from isolated type species, no MAG sequences from external databases were considered in this phylogenomic study. The AAI percentages of the reconstructed HQ MAGs M2_1C_046 and M3_2C_046 were equal or lower than 52.2% for all studied species of the family *Cyclobacteriaceae* and 50.3% between them. These values were below the results of the other representative species of the other genera of the family *Cyclobacteriaceae* among themselves (Figure S5). In addition, orthoANI values were lower than the threshold for genus and species delineation [68] (Figure S5). The core genome tree based on 906 concatenated orthologous sequences translated to proteins of the two HQ MAG and 19 representative species from the family *Cyclobacteriaceae* (Figure 5) support the establishment of MAGs M2_1C_046 and M3_2C_046 as two new species within two different genera within this family, for which we propose the names “*Candidatus* Salsimicrobium terrae gen. nov., sp. nov. and “*Candidatus* Terripaludimicrobium onubense gen. nov., sp. nov., respectively.

#### 3.3.5. Uncovered Diversity of the Family *Balneolaceae*

The HQ MAG M3_1C_030 (3,149,191 bp; 43.64 mol% G+C) was identified as a member of the genus *Halalkalibaculum*, within the family *Balneolaceae* (Table S2). The phylum *Balneolota*, that harbors *Balneolaceae*, is one of the most predominant phyla in hypersaline soils and sediments [16,17,18,19]. Currently, the family *Balneolaceae* includes five genera: *Balneola*, *Fodinibius*, *Gracilimonas*, *Halalkalibaculum* and *Rhodohalobacter*. Moreover, a species from the family *Balneolaceae* was recently isolated and characterized from the soils of this study, *Fodinibius salsisoli* [14]. The highest number of identified HQ and MQ MAGs within this study were assigned to these two genera, i.e., 14 and 6 MAGs for *Halalkalibaculum* and *Fodinibius*, respectively. Additionally, one MAG was assigned to the genus *Gracilimonas*, and five of them were identified to the family level. With a total of 26 MAGs, the family *Balneolaceae* is the taxonomic family with the highest number of high- and medium-quality reconstructed sequences in this analysis (Figure S1).

**Figure 6:**
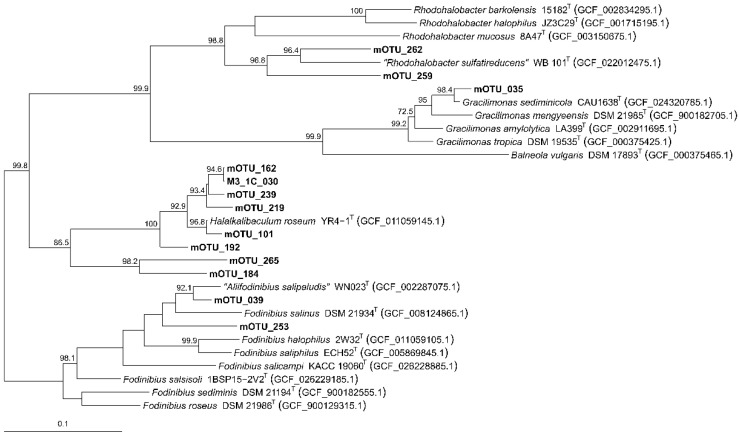
Approximately maximum-likelihood phylogenomic tree based on 155 concatenated orthologous translated proteins of M3_1C_030, mOTUs clustered from all the MAGs assigned to the family *Balneolaceae*, and the representative species from this family. Bootstrap values above 70% are shown above the branches. Bar, 0.1 substitutions per nucleotide position.

Due to the high representation of the family *Balneolaceae* in the environment under study, an in-depth analysis of all the reconstructed MAGs related to this family was performed instead of restricting it to the HQ MAGs. In total, 51 MAGs (including high-, medium- and low-quality sequences) were clustered into 12 mOTUs based on ANI and quality, which were identified as members within the genera *Fodinibius*, *Halalkalibaculum*, *Gracilimonas*, as well as unknown taxa (Table S3). Due to the medium-to-low quality of the MAGs constituting the mOTUs, these showed identities under 90.3% AAI and 92.0% ANI among themselves. Exceptionally, mOTU_162 showed values close to 100% for both indexes (98.9% AAI and 98.6% orthoANI) with HQ MAG M3_1C_030 (Figure S6). These results, along with the close relationship between them shown in the core genome tree based on the 155 concatenated orthologous sequences translated to proteins from the sequences from this study and 18 genomes of representative species of the genera of the family *Balneolaceae* (Figure 6), indicate that mOTU_162 and HQ MAG M3_1C_030 constitute a single new taxon within the genus *Halalkalibaculum*.

Besides the aforementioned mOTU_162, several of the clustered mOTUs (i.e., mOTU_101, mOTU_239, mOTU_162, mOTU_219 and mOTU_192) are also closely related to the latest genus characterized within the family *Balneolaceae*, *Halalkalibaculum* (Figure 6). They show AAI values equal or higher than 85.3% with *Halalkalibaculum roseum*, higher than the 65─72% cutoff for genus delineation [66,67], but the orthoANI percentages were equal or lower than 80.4%, below the 95% threshold for delineation of species [63,64,65]. These results indicate that they are indeed members of the genus *Halalkalibaculum*, different to the species *H. roseum* (Figure S6). Furthermore, they seem to constitute at least five uncovered species of the genus *Halalkalibaculum*. Similarly, mOTU_039 and mOTU_253 seem to constitute two new species within the genus *Fodinibius* (65.9−80.8% AAI; 70.3−80.3 and 69.6−70.8% orthoANI, respectively); mOTU_262 and mOTU_259, two new species within the genus *Rhodohalobacter* (63.5−76.2 and 62.8−64.9% AAI; 68.5−74.6 and 68.9−69.7% orthoANI, respectively); and mOTU_035, a new species within the genus *Gracilimonas* (70.1−88.3% AAI, 71.9−86.8% orthoANI). On the other hand, mOTU_184 and mOTU_265 cluster together in a clearly separate branch from the genus *Halalkalibaculum* and its closely related mOTUs (Figure S6), and their OGRIs values are low against all the representative species of the family *Balneolaceae* (Figure 6). Thus, these two mOTU are constituting two different species in a putative, not-yet-described genus from the family *Balneolaceae*. All these results seem to indicate that these mOTUs constitute new species and genera within the family. As the sequences do not meet high-quality requirements [35], we merely propose this possibility but do not formally designate them as *Candidatus* taxa.

To conclude, the family *Balneolaceae* is one of the better represented families in the hypersaline soils of the Odiel Saltmarshes Natural Area. All the MAGs reconstructed in this study constituted new species within known genera of this family, specially, the genus *Halalkalibaculum*. Furthermore, two of the mOTU clusters seem to establish their own genus, without any representative isolate to date. Hence, this family harbors numerous species which have not been isolated or characterized yet. In this study, we propose a new Candidatus species within the genus *Halalkalibaculum* based on the high-quality reconstructed MAG M3_1C_030, for which we propose the name “*Candidatus* Halalkalibaculum distributum” sp. nov.

### 3.4. Strategies to Survive Extreme Conditions Coded in HQ MAG Sequences

#### 3.4.1. Transporters and Biosynthesis Routes to Deal with Salinity

**Figure 7:**
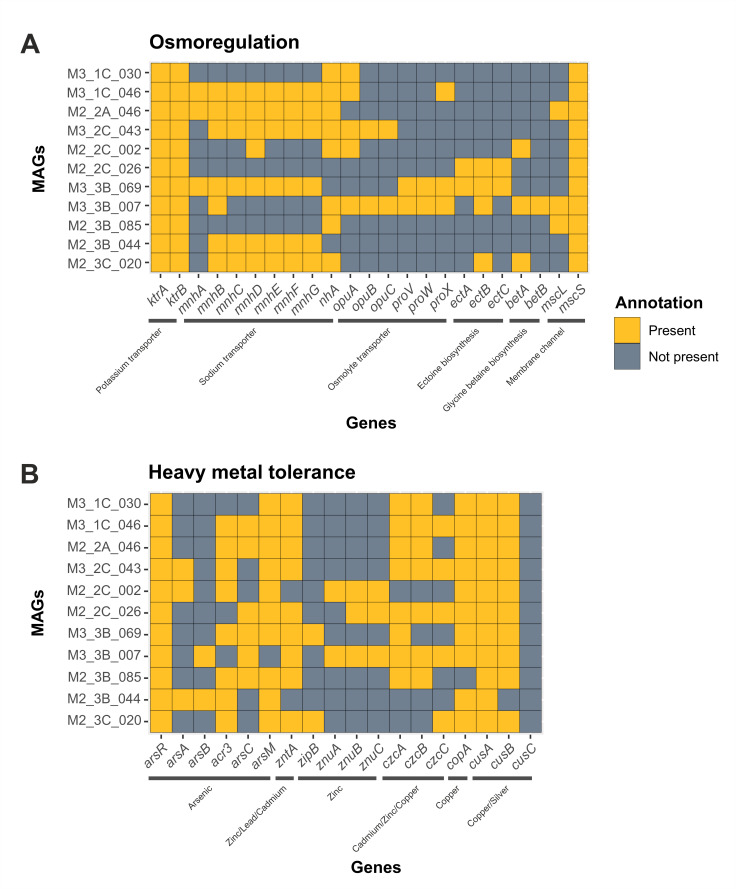
Presence (gold) or absence (gray) of genes related to (**A**) osmoregulation and (**B**) heavy metal tolerance mechanisms in the 11 HQ MAGs.

Osmoregulatory mechanisms are essential for inhabiting hypersaline environments. First barrier of defense against osmotic shock includes KtrAB potassium importers, which contribute to osmotic balance, and Mnh and NhaA sodium importers, which protect against sodium toxicity [72,73,74,75]. KtrAB was identified in the proteome of all 11 HQ MAGs, along with at least one sodium efflux-related function, except for M3_3B_026, which lacked both Mnh and NhaA (Figure 7A; Table S4). Furthermore, KtrAB is the only sodium-dependent potassium importer, indicating its relevance in osmoprotection [76,77] and its role in the metabolism of our MAGs.

In addition, osmolytes can be transported into the cytoplasm for long-term adaptation to salt stress by OpuABC, which preferentially transports choline [78,79], and OpuD and ProVWX, with preference for glycine betaine [80,81]. The *opuABC* operon was detected exclusively in M2_2C_043 and M2_2C_007 (the only MAG identified at the species level as *Pseudomonas teatrolens*), whereas *proVWX* was also found in M2_2C_007 as well as M3_3B_069. Thus, osmolyte uptake from the environment seems to be uncommon among our 11 HQ MAGs. Likewise, genes associated with the universal biosynthesis of osmolytes, including *ectABC* for ectoine and *betAB* for glycine betaine, were annotated in M2_2C_026, M2_3C_069 and M2_2C_007. The absence of osmolite transport and biosynthesis in M3_1C_030 was also found in other members of the family *Balneolaceae*, particularly in *Fodinibius salsisoli*, a recently described species isolated from the environment under study [14].

To sum up, the 11 MAGs with highest quality reconstructed in this study sequences related to regulatory mechanisms for ion transport under salt stress situations, but only a few of them showed functions related to the use of osmolites for osmoregulation (i.e., osmolites transport and biosynthesis of universal compatible solutes, such as ectoine and glycine betaine).

#### 3.4.2. Extruding and Detoxification of Heavy Metals

Mining and industrial activities have resulted in the pollution of water and sediments of the Odiel River, with concentrations over the recommended limit for arsenic, lead and zinc, among other heavy metals [21,29,30]. The studies of the functional profile of the prokaryotic community inhabiting the hypersaline soils of the Odiel Saltmarshes Natural Area have revealed the presence of genes related to heavy metal tolerance [12,13].

The annotation of predicted coding proteins in the 11 HQ MAGs against KEGG database showed that mechanisms related to arsenic tolerance are not widely distributed. Most MAGs did not encode the *arsAB* arsenic transporter responsible for exporting arsenite from the cytoplasm to the extracellular medium. However, the majority encoded the *acr3* transporter, consistent with the overall community profile, where *acr3* was more prevalent than *arsA* and *arsB* [21]. The regulatory protein ArsR was found in the 11 MAGs. On the other hand, ArsC catalyzes the reduction of arsenate to arsenite, which is extruded outside the cell by aforementioned arsenic transporters. This protein was identified in the HQ MAGs identified as M3_1C_046, M2_2A_046, M3_3B_026, M2_3C_069, M2_2C_007 and M3_3B_085 (Figure 7B). On the other hand, M3_1C_030 did not reveal any functions related to arsenic tolerance, in accordance with the species from the family *Balneolaceae*, isolated for the first time from the hypersaline soils of the Odiel Saltmarshes Natural Area, i.e., *Fodinibius salsisoli* [14].

The S-adenosylmethionine methyltransferase, ArsM, transforms inorganic arsenic into organoarsenic compounds. This enzyme is widespread among prokaryotes [82] and it has been annotated in contigs related to the most abundant phyla in the samples under study, particularly, in the phylum *Methanobacteriota* and in the genomes of species isolated from this environment [12,13,14]. This enzyme sequence is found in the proteome of M3_1C_030, M2_1C_046, M3_2C_046, M2_2A_002, M3_3B_026, M3_3B_085, M2_3B_044, M2_3B_020, and M2_3C_069. Hence, its presence suggests that this function has a relevant role for arsenic tolerance even if other mechanisms are not present.

Zinc, lead and cadmium P-type ATPase transporter, ZntA [83,84,85,86] is the most abundant function annotated against the KEGG database for the prokaryotic population of the Odiel Saltmarshes Natural Area [21]. It was identified in the predicted proteins of all HQ MAGs, except for M2_2A_002 and M2_3B_044. Zinc uptake functions, such as *zipB* or *znuABC* [86,87], were not encoded in most of the HQ MAGs. Cadmium, zinc and copper efflux system CzcCBA [88,89] was present in M3_1C_030, M2_1C_046, M3_2C_046, M2_2C_043, M3_3B_026, M2_2C_007 and M3_3B_085. CopA is a relevant copper exporter [90] and its coding sequences have been identified in all the HQ MAGs with the exception of M3_3B_085. Furthermore, two of the genes encoding the copper/silver efflux system CusABC [91] were identified for the 11 HQ MAGs (Figure 7B; Table S4). Thus, the genomic sequences show that the taxa harbor mechanisms to extrude the overflow of heavy metal elements.

To sum up, the 11 HQ MAGs present coding sequences related to cadmium, zinc and copper tolerance. Similarly, *copA, zntA, czcCBA* and *cusAB* have also been identified in the genome of isolates of novel species from this environment [12,13]. On the other hand, functions related to arsenic tolerance are not as well represented among the bacterial MAGs.

### 3.5. A Glimpse into the Metabolic Activity of the Novel Candidatus Taxa

*Wenzhouxiangella* (family *Wenzhouxiangellaceae*) is one of the dominant genera of *nirS*-type denitrifying bacteria from ponds [92]. Nitrate reductase (Nir) is a key enzyme in the denitrification process, an indispensable part of the nitrogen cycle [93,94]. It is constituted by *nirK* and *nirS* isozymes [95,96,97]; both of them are present in the assembled sequences of the MAG related to *Wenzhouxiangellaceae*, as well as the HQ MAG related to the phylum *Nitrospinota* (M3_3B_026).

The few species isolated to date within the phylum *Gemmatimonadota* exhibit intense pigmentation due to the presence of multiple, and mostly uncharacterized, carotenoids [98]. M2_2A_002 harbored *cruC, crtB* and *crtI* genes related to carotenoid biosynthesis in its genomic sequence. Thus, reddish pigmentation should be expected from the colonies of this currently uncultured taxon. In addition, the genes coding menaquinone (vitamin K2) biosynthesis pathway [99] were complete *(menABCDEFH* and *ubiE*) with the exception of *menI*.

The metabolism encoded by the genomic sequences of the two MAGs related to the family *Cyclobacteriaceae* (M3_2C_046 and M2_1C_046) present differences. On one hand, M3_2C_046 harbors genes related to the biosynthesis of arginine (*argABCDEFGH*) and genes coding for the hydrogen dehydrogenease enzyme (*hoxF*, *hoxU*, *hoxY* and *hoxH*) [37], whereas M2_1C_046 presents genes related to the high-costing biosynthesis of biotin (i.e., *bioA*, *bioD*, *bioB*, *fabF*, *fabG* and *fabZ*), with only *bioH* and *fabI* missing. The differences in their metabolism, especially of high conserved routes such as biotin biosynthesis, highlight the taxonomic distances among these two species.

Previously, genes related to biotin biosynthesis had been identified in the genome of the isolates of the species of the family *Balneolaceae* [14]. This vitamin is a costly metabolite that most organisms acquire from an exogenous source [100]. However, five HQ MAGs (M3_1C_030, M2_1C_046, M2_2C_043, M2_2C_007 and M2_3B_020) showed functions related to the first stage of the biotin biosynthesis, particularly, the BioC-BioH pathway (i.e., *fabF*, *fabG*, *fabZ*, *fabI*) [101,102]. For the second and better-characterized part of the route, genes *bioF, bioA, bioD* and bioB were identified. The *bioC* gene was missing in some members of the family *Balneolaceae* too, and *bioH* was not detected in any species of this family [14]. However, the *bioH* gene has multiple homologs, making its annotation against public databases challenging [101,103,104,105,106]. Regardless, the near-complete presence of this route clearly indicates that genes related to biotin biosynthesis are harbored in the genome of these four putative new species from the hypersaline soils of the Odiel Saltmarshes Natural Area.

Oxidative nitrate activity has been observed in the phylum *Nitrospinota* [107,108,109,110]. Genes related to nitrogen assimilation, particularly, *nifBDEHKN*, *nirB*, *nirK* and *nirS*, were annotated in M3_3B_026. Kop et al. [107] detected the *nirK* gene in most high-quality genomes related to this phylum. However, *nifBDEHKN* and *nirS* are absent from the class *Nitrospinia*, the only class described for *Nitrospinota*. This could indicate that M3_3B_026 is phylogenetically distant to other putative species of the phylum that have near-complete genomes. As previously stated, *nirK* and *nirS* constitute the Nir isoenzyme [95,96,97], whereas *nifBDEHKN* are related to nitrogen fixation [111], a process that transforms atmospheric nitrogen gas (N2) into ammonia [112]. This conversion is essential to the bio-availability of nitrogen, which cannot be assimilated as N_2_ by most organisms [113].

## 4. Conclusions

The hypersaline soils of the Odiel Saltmarshes Natural Area represent an extreme environment inhabited by a diverse and unexplored prokaryotic population. Its complexity obscures the reconstruction of high-quality MAGs. However, in this study, 11 HQ MAGs were recovered and analyzed from 18 shotgun metagenomic samples, out of more than 4000 total MAG sequences. Moreover, with the exception of M2_2C_007, identified as *Pseudomonas taetrolens*, the HQ MAGs were not closely related to known taxa. The comparative genomic analysis based on the core proteome inference and the OGRIs of high-quality sequences determined six novel taxa within five different bacterial phyla (*Pseudomonadota*, *Gemmatimonadota*, *Nitrospinota*, *Bacteroidota* and *Balneolota*) and predicted several more.

The in-depth analysis of survival mechanisms under extreme salt and heavy metal concentration showed functions related to ion transport. However, most MAGs lacked functions associated with the biosynthesis and/or transport of well-known osmolytes for long-term osmoregulation. Additionally, cadmium, zinc, and copper tolerance genes were observed, but no evidence of arsenic metabolism was detected. In addition, nitrogen fixation activity was found in M2_3B_020 and M3_3B_026. M2_2A_002 harbored genes related to carotenoid biosynthesis, M3_2C_046 for arginine biosynthesis and multiple MAGs for the high-cost biosynthesis of biotin (M3_1C_030, M2_1C_046, M2_2C_043, M2_2C_007 and M2_3B_020). These findings highlight hypersaline soils as a promising source of microorganisms with significant biosynthetic potential for biotechnological applications.

Below, we include the descriptions of the new taxa: four new *Candidatus* genera (represented by MAGs M2_2A_002, M3_3B_026, M2_1C_046 and M3_2C_046) and six new *Candidatus* species (represented by MAGs M2_3B_020, M2_2A_002, M3_3B_026, M2_1C_046, M3_2C_046 and M3_1C_030).

**Description of “*Candidatus* Wenzhouxiangella saliterrae” sp. nov.**

“*Candidatus* Wenzhouxiangella saliterrae” sp. nov. (sa.li.ter’rae. L. masc. n. *sal*, salt; L. fem. n. *terra*, soil; N.L. gen. n. *saliterrae*, of saline soil.).

It was reconstructed from hypersaline soils at the saltmarshes of the Odiel Natural Park in Huelva (Southwest Spain). It belongs to the family *Wenzhouxiangellaceae*, order *Chromatiales*, class *Gammaproteobacteria*, and phylum *Pseudomonadota*. Its genome has an approximate size of 3.60 Mb, its G+C content is 66.5 mol%, and it is available at the NCBI BioSample repository with accession SAMN46541416 (M2_3B_020).

**Description of “*Candidatus* Terrihalomicrobium” gen. nov.**

“*Candidatus* Terrihalomicrobium” gen. nov. (Ter.ri.ha.lo.mi.cro’bi.um. L. fem. n. *terra*, soil; Gr. masc. n. *hals*, salt; N.L. neut. n. *microbium*, microbe; N.L. neut. n. *Terrihalomicrobium*, a microbe from salty soil).

This genus belongs to the order *Longimicrobiales*, class *Longimicrobiia*, and phylum *Gemmatimonadota*. It is proposed as a new genus because its sequence similarity to the closest related species is below the accepted threshold for genus delineation..

**Description of “*Candidatus* Terrihalomicrobium hispanicum” sp. nov.**

“*Candidatus* Terrihalomicrobium hispanicum” sp. nov. (his.pa’ni.cum. L. neut. adj. *hispanicum*, from Spain).

It was reconstructed from hypersaline soils at the saltmarshes of the Odiel Natural Park in Huelva (Southwest Spain). Its genome has an approximate size of 3.35 Mb, its G+C content is 70.4 mol%, and it is available at the NCBI BioSample repository with accession SAMN46543193 (M2_2A_002).

**Description of “*Candidatus* Nitrohalomicrobium” gen. nov.**

“*Candidatus* Nitrohalomicrobium” gen. nov. (Ni.tro.ha.lo.mi.cro’bi.um. L. neut. n. *nitrum*, native soda, *natron*, nitrate; Gr. masc. n. *hals*, salt; L. masc. dim. n. *microbium*, a microbe; N.L. masc. n. *Nitrohalomicrobium*, halophilic nitrate microbe).

This genus is placed within the phylum *Nitrospinota*, and it is proposed as a new genus because its sequence similarity to the closest related species is below the accepted threshold for genus delineation. 

**Description of “*Candidatus* Nitrohalomicrobium salipaludis” sp. nov.**

“*Candidatus* Nitrohalomicrobium salipaludis” sp. nov. (sa.li.pa.lu’dis. L. masc. n. *sal* (gen. *salis*), salt; L. gen. fem. n. *paludis*, of a swamp; N.L. gen. fem. n. *salipaludis*, of a saltmarsh).

It was reconstructed from hypersaline soils at the saltmarshes of the Odiel Natural Park in Huelva (Southwest Spain). Its genome has an approximate size of 2.85 Mb, its G+C content is 61.2 mol%, and it is available at the NCBI BioSample repository with accession SAMN46543291 (M3_3B_026).

**Description of “*Candidatus* Salsimicrobium" gen. nov.**

“*Candidatus* Salsimicrobium” gen. nov. (Sal.si.mi.cro’bi.um. L. masc. perf. part. *salsus*, salted; N.L. neut. n. *microbium*, microbe; N.L. neut. n. *Salsimicrobium*, a salted microbe).

This genus belongs to the family *Cyclobacteriaceae*, order *Cytophagales*, class *Cytophagia*, and phylum *Bacteroidota*. It is proposed as a new genus because its sequence similarity to the closest related species is below the accepted threshold for genus delineation..

**Description of “*Candidatus* Salsimicrobium terrae" sp. nov.**

“*Candidatus* Salsimicrobiun terrae” sp. nov. (ter’rae. L. gen. n. *terrae*, of soil).

It was reconstructed from hypersaline soils at the saltmarshes of the Odiel Natural Park in Huelva (Southwest Spain). It belongs to the family *Cyclobacteriaceae*, order *Cytophagales,* class *Cytophagia*, and phylum *Bacteroidota*. Its genome has an approximate size of 4.75 Mb, its G+C content is 37.9 mol%, and it is available at the NCBI BioSample repository with accession SAMN46545946 (M2_1C_046).

**Description of “*Candidatus* Terripaludimicrobium” gen. nov.**

“*Candidatus* Terripaludimicrobium” gen. nov. (Ter.ri.pa.lu.di.i.cro’bi.um. L. fem. N. *terra*, soil; L. fem. N, *palus* (gen. *paludis*), a swamp, marsh; N.L. neut. N. *microbium*, microbe; N.L. neut. N. *Terripaludimicrobium*, a microbe from marsh soil).

This genus belongs to the family *Cyclobacteriaceae*, order *Cytophagales*, class *Cytophagia*, and phylum *Bacteroidota*. It is proposed as a new genus because its sequence similarity to the closest related species is below the accepted threshold for genus delineation.i

**Description of “*Candidatus* Terripaludimicrobium onubense” sp. nov.**

“*Candidatus* Terripaludimicrobium onubense” sp. nov. (o.nu.ben’se. L. neut. adj. *onubense*, of or belonging to Onuba, currently Huelva).

It was reconstructed from hypersaline soils at the saltmarshes of the Odiel Natural Park in Huelva (Southwest Spain). Its genome has an approximate size of 6.25 Mb, its G+C content is 40.1 mol%, and it is available at the NCBI BioSample repository with accession SAMN46545915 (M3_2C_046).

**Description of “*Candidatus* Halalkalibaculum distributum” sp. nov.**

“*Candidatus* Halalkalibaculum distributum” (dis.tri.bu’tum. L. neut. adj. *distributum*, distributed [widely]).

It was reconstructed from hypersaline soils at the saltmarshes of the Odiel Natural Park in Huelva (Southwest Spain). It belongs to the family *Balneolaceae*, order *Balneolales*, class *Balneolia*, and phylum *Balneolota*. Its genome has an approximate size of 3.15 Mb, its G+C content is 43.6 mol%, and it is available at the NCBI BioSample repository with accession SAMN46545988 (M3_1C_030).

## Figures and Tables

**Table 1:** Metagenomic datasets used in this study and their associated metadata. EC, electrical conductivity.

Sample	Area	Coordinates	Year	EC (mS/cm)	No. Total Reads	Accession Number
M3_1A	1	37°12′26.6″ N 6°57′52.5″ W	2021	27.0	174,068,320	SRS20604428
M3_1B	1	37°12′26.6″ N 6°57′52.5″ W	2021	26.9	174,568,698	SRS20617304
M3_1C	1	37°12′26.6″ N 6°57′52.5″ W	2021	22.4	136,267,104	SRS20617300
M3_2A	2	37°12′28.4″ N 6°57′27.9″ W	2021	24.7	159,093,934	SRS20617299
M3_2B	2	37°12′28.4″ N 6°57′27.9″ W	2021	24.0	143,483,116	SRS20604433
M3_2C	2	37°12′28.4″ N 6°57′27.9″ W	2021	18.5	150,290,774	SRS20617296
M3_3A	3	37°13′18.0″ N 6°57′44.8″ W	2021	23.3	159,330,400	SRS20617298
M3_3B	3	37°13′18.0″ N 6°57′44.8″ W	2021	21.9	171,035,056	SRS20604749
M3_3C	3	37°13′18.0″ N 6°57′44.8″ W	2021	15.4	169,234,708	SRS20604748
M2_1A	1	37°12′26.6″ N 6°57’52.5″ W	2020	33.5	167,417,500	SRS20604429
M2_1B	1	37°12′26.6″ N 6°57′52.5″ W	2020	42.6	172,740,278	SRS20617295
M2_1C	1	37°12′26.6″ N 6°57′52.5″ W	2020	27.1	166,186,480	SRS20617297
M2_2A	2	37°12′28.4″ N 6°57′27.9″ W	2020	46.1	162,525,972	SRS20617301
M2_2B	2	37°12′28.4″ N 6°57′27.9″ W	2020	46.0	106,685,200	SRS20604431
M2_2C	2	37°12′28.4″ N 6°57′27.9″ W	2020	37.9	111,532,290	SRS20604430
M2_3A	3	37°13′18.0″ N 6°57′44.8″ W	2020	39.1	125,176,826	SRS20617303
M2_3B	3	37°13′18.0″ N 6°57′44.8″ W	2020	60.7	191,221,338	SRS20617302
M2_3C	3	37°13′18.0″ N 6°57′44.8″ W	2020	69.2	152,468,274	SRS20604432

**Table 2:** Total number of Metagenome-Assembled Genomes (MAGs) classified by sample and following the MIMAG criteria (HQ, high-quality; MQ, medium-quality; LQ, low-quality).

Sample	HQ	MQ	LQ	Total
M3_1A	0	8	203	288
M3_1B	0	16	208	307
M3_1C	1	7	205	295
M3_2A	0	27	153	289
M3_2B	0	22	107	212
M3_2C	1	26	125	222
M3_3A	0	11	175	259
M3_3B	2	21	172	303
M3_3C	0	10	167	263
M2_1A	0	8	241	353
M2_1B	0	10	180	277
M2_1C	1	13	144	246
M2_2A	1	19	155	258
M2_2B	0	20	136	226
M2_2C	2	20	153	256
M2_3A	0	12	148	243
M2_3B	2	8	126	216
M2_3C	1	15	121	205
Total	11	273	2919	4718

## Data Availability

Data from this article can be found online at PRJNA1227418.

## Supplementary Materials

The following supporting information can be downloaded at: https://www.mdpi.com/article/10.3390/microorganisms14020489/s1, Figure S1: Distribution at family rank of the 286 HQ and MQ MAGs reconstructed from the 18 metagenomes from the hypersaline soils of the Odiel Saltmarshes Natural Area, Figure S2: AAI and orthoANI values between the MAG M2_3B_020 and other species of the family *Wenzhouxiangellaceae*, Figure S3: AAI and orthoANI values between the MAG M2_2A_002 and other species and MAGs of the phylum *Gemmatimonadota*, Figure S4: AAI and orthoANI values between the MAG M3_3B_026 and other representative species and MAGs of the phylum *Nitrospinota*, Figure S5: AAI and orthoANI values between the MAG M2_1C_046 and M3_2C_046 and other species of the family *Cyclobacteriaceae*, Figure S6: AAI and orthoANI values between M3_1C_030, mOTU_035, mOTU_039, mOTU_101, mOTU_162, mOTU_184, mOTU_192, mOTU_219, mOTU_239, mOTU_253. mOTU_259, mOTU_262 and mOTU_265, as well as other representative species of the family *Balneolaceae*, Table S1: Features of the DNA sequences pre- and post-processing. Table S2: Features of the 11 high-quality MAGs reconstructed from the 18 metagenomic datasets, Table S3: mOTU clustered from medium- and low-quality MAGs identified as members of the family *Balneolaceae*, Table S4: KO identifiers, definitions, and categories, for studied genes in the 11 HQ MAGs.

## Abbreviations

The following abbreviations are used in this manuscript:

AAI	Average Amino acid Identity
ANI	Average Nucleotide Identity
bp	Base pairs
Ca.	Candidatus
EC	Electrical conductivity
FPM	Features Per Million
GTDB	Genome Taxonomy Database
HQ	High quality
ICNP	International Code of Nomenclature of Prokaryotes
KEGG	Kyoto Encyclopedia of Genes and Genomes
KO	KEGG Orthology
LQ	Low quality
MAG	Metagenome-Assembled Genome
Mb	Megabases
MDPI	Multidisciplinary Digital Publishing Institute
MIMAG	Minimum Information about a Metagenome-Assembled Genome
mOTUs	metagenomic Operative Taxonomic Units
MQ	Medium quality
mS/cm	millisiemens per centimeter
OGRIs	Overall Genome Relatedness Indexes
orthoANI	Average Nucleotide Identity for orthologous sequences
