# Supplementary figures and images for: Uncovering the Prokaryotic Diversity of Hypersaline Soils of Odiel Saltmarshes Natural Area Through Metagenome-Assembled Genomes
Source: Microorganisms. 2026 Feb 18;14(2):489. doi: 10.3390/microorganisms14020489 (PMC12942990; doi:10.3390/microorganisms14020489)

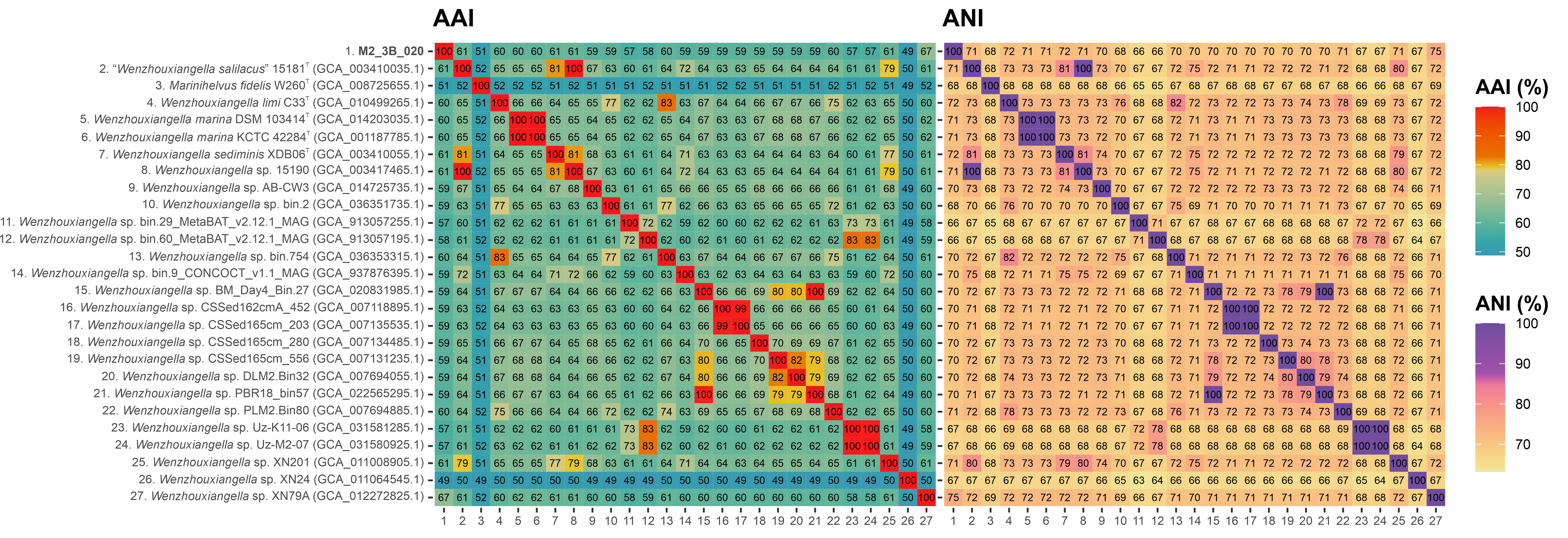

Supplement: Supplementary file 1 [file microorganisms-14-00489-s001.zip › Figure_S2.tif]

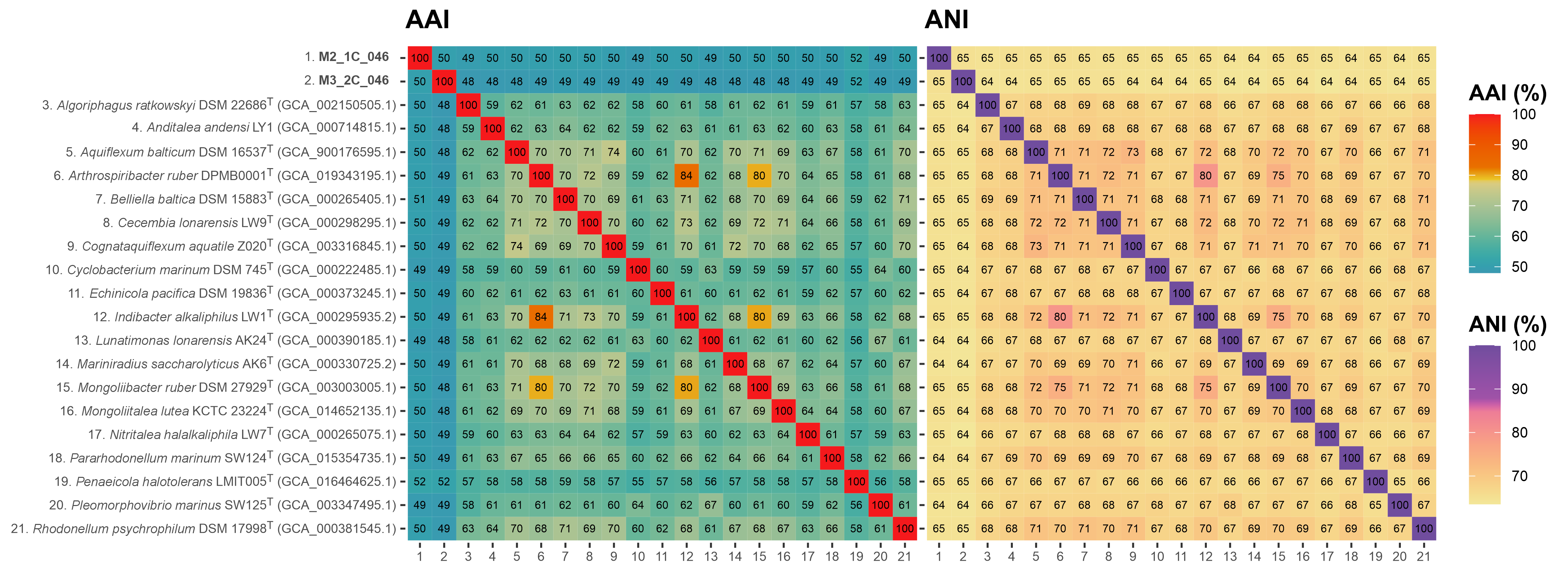

Supplement: Supplementary file 1 [file microorganisms-14-00489-s001.zip › Figure_S5.tif]

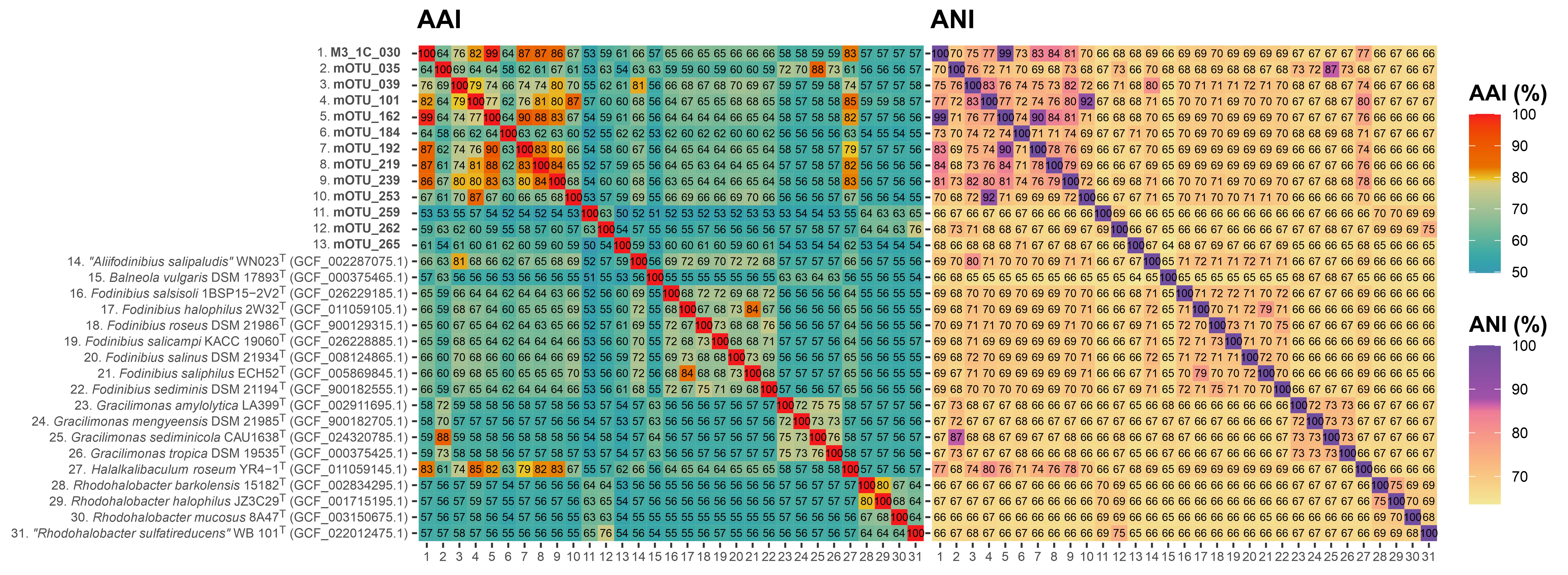

Supplement: Supplementary file 1 [file microorganisms-14-00489-s001.zip › Figure_S6.tif]
